# Tolerability of overnight rotigotine transdermal patch combined with intrajejunal levodopa infusion at 1 year: a 24-h treatment option in Parkinson’s disease

**DOI:** 10.1007/s00702-022-02506-4

**Published:** 2022-05-03

**Authors:** Yue Hui Lau, Valentina Leta, Katarina Rukavina, Miriam Parry, Jenny Ann Natividad, Vinod Metta, Guy Chung-Faye, K. Ray Chaudhuri

**Affiliations:** 1 Department of Basic and Clinical Neurosciences, The Maurice Wohl Clinical Neuroscience Institute, Institute of Psychiatry, Psychology & Neuroscience, King’s College London, London, SE5 9RT UK; 2 Parkinson’s Foundation Centre of Excellence, King’s College Hospital, London, UK; 3 Department of Gastroenterology, King’s College London, King’s College Hospital NHS Foundation Trust, London, UK; 4 King’s College Hospital London, Dubai, UAE

**Keywords:** Tolerability, Parkinson’s disease, Dopamine agonists, Rotigotine transdermal patch, Intrajejunal levodopa infusion, Personalised medicine

## Abstract

**Background:**

Twenty-four-hour treatment options could provide a continuous drug delivery strategy in advanced Parkinson’s disease and can ameliorate motor and non-motor complications. Use of levodopa infusion is often limited to 12–16 h/day due to its cost. Adjunctive overnight rotigotine transdermal patch is a continuous drug delivery option successfully used in clinical practice coupled with apomorphine infusion. However, real-life data addressing the tolerability of transdermal dopamine agonist therapy with concomitant use of intrajejunal levodopa infusion in advanced Parkinson’s disease are not available.

**Objective:**

To evaluate the tolerability and beneficial effects of combined therapy with overnight rotigotine transdermal patch and intrajejunal levodopa infusion over a follow-up period of 12 months in advanced Parkinson’s disease.

**Method:**

In this retrospective data analysis, data before and after the initiation of the continuous drug delivery combined therapy using overnight rotigotine transdermal patch and intrajejunal levodopa infusion were collected from the ongoing non-motor-international-longitudinal study (NILS) and local clinical practice at King’s College Hospital (London, United Kingdom). 12 advanced Parkinson’s disease patients on intrajejunal levodopa therapy who were additionally treated with overnight rotigotine transdermal patch (mean dose 5.67 ± 4.19 mg) are included. Tolerability over a 12-month period was assessed. In addition, changes in motor symptoms (SCales for Outcomes in Parkinson's disease, SCOPA-Motor), non-motor symptoms (Non-Motor Symptoms Scale, NMSS) and quality of life (Parkinson's disease Questionnaire-8, PDQ-8) before and 12-month after continuous drug delivery combined therapy initiation are evaluated.

**Results:**

Tolerability was 100% irrespective of age, disease duration, stages of disease. (Treatment with overnight rotigotine transdermal patch that was maintained for a minimum of 6 months was considered “tolerated”, primary tolerability). In addition, we noted a significant reduction of the NMSS total score (*p* = 0.009) and the NMSS domain 3 score (mood and apathy domain) (*p* = 0.028), although the latter did not remain statistically significant after correction for multiple testing (*p*2 = 0.252) at 12 months.

**Conclusion:**

Combination of intrajejunal levodopa infusion with overnight rotigotine transdermal patch is well tolerated and extend the beneficial effects of infusion with excellent tolerability; and also improved aspects of mood and apathy sustained at 12 months in advanced Parkinson’s disease.

## Introduction

In advanced Parkinson’s disease (PD), the progressive degeneration of dopaminergic neurons combined with pulsatile dopaminergic stimulation increase the vulnerability to motor and non-motor complications (Timpka et al. 2016; Rukavina et al. 2021) Several clinical and experimental observations have shown that continuous drug delivery (CDD) is associated with fewer motor and non-motor complications, as compared to pulsatile stimulation in advanced PD (Ray Chaudhuri et al. 2016; van Wamelen et al. 2018; Olanow et al. 2020; Leta et al. 2019).

CDD utilising a non-oral approach is beneficial given the potential “blocks” to oral therapies related to PD and comorbid upper gastrointestinal tract dysfunction (Ray Chaudhuri et al.^2016^; Metta et al. 2021). Both levodopa and apomorphine infusion therapy have been proven to be effective over the infusion period (Dafsari et al. 2019; Antonini et al.^2017^). However, infusion therapies are costly and often in many countries cannot be prescribed for 24 h even though logistically this may be sensible. Data suggest that strategies to prolong the action of infusion therapy such as intrajejunal levodopa infusion (IJLI) with a catechol-O-methyltransferase (COMT) inhibitor may lead to cost savings while maintaining optimal clinical care (Leta et al. 2020).

Rotigotine transdermal patch has been in clinical use for almost 15 years now. It is an easily administered therapy that can be used in conjunction with infusion therapies such as apomorphine to improve night time symptoms when the infusions are typically stopped; and good tolerability also has been reported (Raeder et al. 2021; Todorova et al. 2013) In this report, we assess the tolerability and beneficial motor an non-motor effects use of the rotigotine transdermal patch overnight in combination with IJLI as a valid therapeutic strategy to provide 24-h treatment, for a follow-up period extending to 12 months. Tolerability was assessed according to the criteria by Shulman et al. (2000) (i.e., treatment with a dopamine agonist that was maintained for a minimum of 6 months was considered “tolerated”, primary tolerability) which were subsequently adopted by Appiah-Kubi et al. (Appiah-kubi et al. 2003; Poewe et al. 2007).

## Methodology

### Study design

This was a retrospective data analysis of a cohort of patients with PD on IJLI and overnight rotigotine transdermal patch who were followed up at the Parkinson’s Foundation Centre of Excellence at King’s College Hospital, London, United Kingdom from 2009 to 2021. To evaluate tolerability and efficacy-related outcomes (motor, non-motor and quality of life), pre and post CDD combined therapy data were collected from the non-motor-international-longitudinal study (NILS) and routine local clinical practice at King’s College Hospital, London, United Kingdom. The history of the concomitant use of the overnight rotigotine transdermal patch and IJLI was noted from the medical records. Locally implemented outcome measures used in the pathway of care for advanced therapies were also analysed where available.

### Patients

Overall, 12 patients from a cohort of 150 patients treated with daily intrajejunal levodopa infusion had been started on a combination of IJLI for 14–16 h per day and overnight rotigotine transdermal patch (8–12 h). The rotigotine transdermal patch is usually applied an hour before stopping the intrajejunal levodopa infusion at night; and the patch will be removed when intrajejunal levodopa infusion is being initiated in the next morning.

All patients included in the study had a diagnosis of idiopathic Parkinson’s disease satisfying the MDS clinical diagnostic criteria for Parkinson's disease (Postuma et al. 2015). There were no changes reported on the various types of medications taken concomitantly by all the patients during the observational period. Patients with advanced dementia, hallucinations or psychosis, severe orthostatic hypotension or history of hallucination and impulse control disorders were excluded; as such patients would not be usually started on dopamine agonist therapy. The dose up-titration was done gradually and usually by 2 mg increments every 2 weeks as per local guidelines at our centre.

### Ethics/audit approvals

Data were collected from the longitudinal non-motor natural history study (The NILS study, UKCRN No 10084) which has received ethical approval from all the related institutions. As this was a clinical observational post-treatment surveillance-based work, using clinically licensed drugs for approved indication and utilizing routinely used clinical assessment tools, the study was registered as a clinical audit at King’s College Hospital, London, United Kingdom.

### Assessments

Demographics and disease-related characteristics assessed included sex, age, disease duration, disease stage (Hoehn and Yahr Scale), total rotigotine transdermal patch dose, total IJLI dose, total levodopa equivalent dose and side effects of rotigotine transdermal patch use. Long term tolerability, side effects and attrition rates were noted in a retrospective manner.

Assessment of motor symptoms (Scopa Motor), Hoehn and Yahr (HY) staging, non-motor symptoms (NMSS) and quality of life (Qol, PDQ-8) were performed. The follow-up assessments were performed at routine clinic visits at 6 months intervals after initiation of individual therapies and data is presented from assessments made at the clinic visit at 12 months for each patient.

### Data analysis

Descriptive statistics (mean and standard deviation, median and range, absolute number and percentages) were obtained for each variable as appropriate. Baseline to follow-up changes were tested using the Wilcoxon signed-ranks test. Benjamini–Hochberg procedure was used to correct for multiple testing. A < 0.05 *p* value was considered statistically significant. Data analyses were performed using Microsoft Excel 2010 and the Statistical Package for the Social Sciences (SPSS), version 25.0 (IBM Corp., Armonk, NY, USA).

## Results

Demographics and disease-related characteristics at baseline are summarised in Table 1. Tolerability was 100% for all patients receiving overnight rotigotine transdermal patch and IJLI. One patient developed pathological gambling while on rotigotine transdermal patch 4 mg, however, the impulse controlled behaviour subsided when the dose was reduced to 2 mg daily. No intrusive side effects related to the use of rotigotine transdermal patch, namely skin reaction, lack of effect, somnolence, hallucination or confusion were noted.

**Table 1:** Main demographics and Parkinson’s disease historical characteristics

Demographic characteristics	All cases (*N* = 12)
Male gender (*n*, %)	6 (50)
Age (years)	67.75 ± 7.58
Disease duration (years)	16.25 ± 5.38
Hoehn and Yahr stage	4 (3–5)
Rotigotine transdermal patch daily dose (mg)	5.67 ± 4.19
IJLI daily dose infusion dose (mg)	59.47 ± 17.93
LEDD	206.97 ± 134.82

Data are presented as mean ± standard deviation, median (range) or number (percentage)

*IJLI* intrajejunal levodopa infusion, *LEDD* l-dopa equivalent daily dose

Changes in motor, non-motor and quality of life-related outcomes are summarised in Table 2. At the end of the observational period (12 months), a significant decrease was observed for the NMSS total score (*p* = 0.009) and the NMSS domain 3 score (mood and apathy domain) (*p* = 0.028), although the latter did not remain statistically significant after correction for multiple testing (*p*2 = 0.252) (Table 2). No further significant changes in outcomes were observed.

**Table 2:** Baseline and 12-month follow-up clinical data

	Baseline		Follow-up		*p*1	*p*2
	Mean	SD	Mean	SD		
SCOPA-motor	30.67	8.53	30.25	10.29	0.695	0.695
NMSS						
Cardiovascular	2.92	3.7	2.75	3.41	0.677	0.885
Sleep/fatigue	14.75	10.68	13.75	7.39	0.666	0.885
Mood/apathy	16.75	19.26	10.08	9.98	0.028	0.252
Perceptual/hallucinations	1.92	3.73	2.58	5.63	0.892	0.892
Attention/memory	11.5	10.71	9.25	6.69	0.168	0.756
Gastrointestinal	8.25	9.85	7.83	9.92	0.858	0.892
Urinary	10.92	8.26	10.67	9.67	0.688	0.885
Sexual functioning	3.27	5.31	2.33	4.66	0.450	0.885
Miscellaneous	13.92	10.27	12.33	8.15	0.408	0.885
Total score	93.17	83.5	71.75	38.44	0.009	0.027
PDQ-8 total score	14.67	6.43	14.17	6.97	0.609	0.695

Data are presented as mean ± standard deviation. Baseline to follow-up changes were tested using the Wilcoxon signed-ranks test. Benjamini–Hochberg procedure was used to correct for multiple testing. A < 0.05 *p* value was considered statistically significant

*SCOPA-Motor* SCales for Outcomes in Parkinson's disease, *NMSS* non-motor symptoms scale, *PDQ-8* Parkinson's disease Questionnaire-8, *p2* corrected *p* value

## Discussion

To our knowledge, this the first report on the tolerability of a combined CDD strategy using overnight rotigotine transdermal patch and IJLI strategy and its beneficial effect on the burden of non-motor symptoms at 12 months follow-up. Rotigotine transdermal patch at maximum tolerated dose 2–10 mg was used in each patient with concomitant use of IJLI.

Our data suggest an excellent and 100% tolerability rates of transdermal DA treatment with concomitant IJLI therapy in a real-life setting at 12 months. In the United Kingdom and in many other countries, IJLI cannot be prescribed more than one cassette a day owing to national policy as well as cost issues (Leta et al. 2020). However, many patients experience a “rebound” when the pump is taken off after a 12–16 h infusion period. Usually, there is also emergence of night-time off periods and fragmentated sleep episodes. Rotigotine transdermal patch is well established as an effective overnight treatment with level 1 evidence reported in post hoc analysis of RECOVER study (Randomized Evaluation of the 24-Hour Coverage: Efficacy of Rotigotine) and works well to deliver a 24-h therapy in an analogous situation when daytime apomorphine infusion is combined with night time rotigotine transdermal patch (Chaudhuri et al. 2013). Data from this review of our IJLI service suggest that rotigotine transdermal patch is similarly beneficial in combination with IJLI therapy and can successfully extend the beneficial period of daily IJLI use with excellent tolerability.

Our observations also suggest that this strategy is useful and can be sustained up to at least 12 months with a significant improvement of non-motor symptoms, possibly driven by improvements in mood and apathy, as compared to baseline. The mood and apathy aspects of non-motor symptoms represent a significant unmet need in relation to treatment of PD. Impulse control disorders were observed in one patient in this cohort during the study period, but it resolved with the reduction of the dose; and clearly clinical monitoring is important.

The noted improvements are in line with observed non-motor symptoms benefits that have been reported with IJLI in the GLORIA and DUOGLOBE database studies as well as rotigotine transdermal patch (Antonini et al. 2017; Chaudhuri et al. 2013; Standaert et al. 2021). The combination of daily apomorphine subcutaneous infusion and rotigotine transdermal patch overnight was safe and useful in the management of PD symptoms, particularly for sleep and mood impairment, at a 2-year follow-up (Todorova et al. 2013) Similarly, the overnight use of opicapone can be effective in controlling night-time symptoms in patients with PD on IJLI, particularly in countries where the use of a single IJLI cassette per day is recommended so that associated costs can be contained (Leta et al. 2020).

The small sample size of our study could also explain the non-statistically significant improvements observed for other NMS-related domains, including the sleep and fatigue domain. Other limitations of this data analysis include the lack of a comparator group. As such, these preliminary pilot data from our report need to be confirmed in larger, controlled and prospective, real-life studies. Such studies are also important as we recognise that treatment related emergent side effects could be underestimated thus showing a skewed tolerability data in a retrospective study such as ours.

Nevertheless, we believe this report has collected important data in relation to good tolerability of overnight rotigotine transdermal patch among patients with IJLI use and for the data collection from all age groups, in which elderly PD patients are frequently being excluded from clinical trials. And therefore, it could be representative of a real-life scenario and clinically implemented in many centres.

Based on our current observational analysis we would recommend that overnight rotigotine transdermal patch should be utilised to deliver 24-h CDD in patient with daytime IJLI. The dose of rotigotine transdermal patch needs to be personalised based on patient age, cognitive capacity as well as skin tolerability but would range from 2 to 10 mg. Overall, the combination of rotigotine transdermal patch and IJLI, with two different pharmacokinetic profiles may enable the use of lower dosages and results in better tolerability and minimized the potential side effects. This becomes particularly useful strategy for patients with advanced PD in the diverse routine clinical care settings as 24-h control of PD symptoms can be difficult.

## Conclusion

In conclusion, this retrospective data analysis shows good tolerability of overnight rotigotine transdermal patch use in PD patients with concomitant use of IJLI. Rotigotine transdermal patch offers a CDD pattern, particularly compared with oral dopaminergic therapies, with a good tolerability profile. The rotigotine transdermal patch has shown a positive effect on the treatment of motor symptoms in mood and apathy, as well as nocturnal sleep and early morning off period. In addition, the rotigotine transdermal patch could be considered as a beneficial option for treating comorbidities of fatigue and reduce fluctuation-related pain. The use of the rotigotine transdermal patch and IJLI treatment in selected patients therefore represents a useful therapeutic strategy to provide an alternative 24-h treatment of PD without having to resort to a 24-h infusion therapy and to provide an overall beneficial effect on NMS and ultimately, quality of life in these patients. In addition, this may guide the delivery of personalised medicine in the advanced stage of PD. It could be a promising therapeutic strategy with potential to reduce the costs associated with this advanced therapy and increase the effectiveness of IJLI therapy.

## Data Availability

The datasets generated during and/or analysed during the current study are available from the corresponding author on reasonable request.

## Declarations

### Publication history

This manuscript is not published before and is not under consideration for publication elsewhere.

## References

[CR1] Antonini A, Poewe W, Chaudhuri KR, Jech R, Pickut B, Pirtošek Z, Szasz J, Valldeoriola F, Winkler C, Bergmann L, Yegin A, Onuk K, Barch D, Odin P, GLORIA Study Co-investigators (2017). Levodopa-carbidopa intestinal gel in advanced Parkinson's: final results of the GLORIA registry. Parkinsonism Relat Disord.

[CR2] Appiah-Kubi L, Nisbet A, Burn DJ (2003). Use and tolerability of cabergoline in young and older people with Parkinson’s disease: a multicentre observational study. J Appl Res.

[CR3] Chaudhuri KR, Martinez-Martin P, Antonini A, Brown RG, Friedman JH, Onofrj M, Surmann E, Ghys L, Trenkwalder C (2013). Rotigotine and specific non-motor symptoms of Parkinson’s disease: post hoc analysis of RECOVER. Parkinsonism Relat Disord.

[CR4] Dafsari HS, Martinez-Martin P, Rizos A, Trost M, Dos Santos Ghilardi MG, Reddy P, Sauerbier A, Petry-Schmelzer JN, Kramberger M, Borgemeester R, Barbe MT, Ashkan K, Silverdale M, Evans J, Odin P, Fonoff ET, Fink GR, Henriksen T, Ebersbach G, Pirtošek Z, EUROPAR and the International Parkinson and Movement Disorders Society Non-Motor Parkinson's Disease Study Group (2019). EuroInf 2: subthalamic stimulation, apomorphine, and levodopa infusion in Parkinson's disease. Mov Disord off J Mov Disord Soc.

[CR5] Leta V, Jenner P, Chaudhuri KR, Antonini A (2019). Can therapeutic strategies prevent and manage dyskinesia in Parkinson's disease? An update. Expert Opin Drug Saf.

[CR6] Leta V, van Wamelen DJ, Sauerbier A, Jones S, Parry M, Rizos A, Chaudhuri KR (2020). Opicapone and levodopa-carbidopa intestinal gel infusion: the way forward towards cost savings for healthcare systems?. J Parkinsons Dis.

[CR7] Metta V, Leta V, Mrudula KR, Prashanth LK, Goyal V, Borgohain R, Chung-Faye G, Chaudhuri KR (2021). Gastrointestinal dysfunction in Parkinson's disease: molecular pathology and implications of gut microbiome, probiotics, and fecal microbiota transplantation. J Neurol.

[CR8] Olanow CW, Calabresi P, Obeso JA (2020). Continuous dopaminergic stimulation as a treatment for Parkinson's disease: current status and future opportunities. Mov Disord off J Mov Disord Soc.

[CR9] Poewe WH, Rascol O, Quinn N, Tolosa E, Oertel WH, Martignoni E, Rupp M, Boroojerdi B, SP 515 Investigators (2007). Efficacy of pramipexole and transdermal rotigotine in advanced Parkinson's disease: a double-blind, double-dummy, randomised controlled trial. Lancet Neurol.

[CR10] Postuma RB, Berg D, Stern M, Poewe W, Olanow CW, Oertel W, Obeso J, Marek K, Litvan I, Lang AE, Halliday G, Goetz CG, Gasser T, Dubois B, Chan P, Bloem BR, Adler CH, Deuschl G (2015). MDS clinical diagnostic criteria for Parkinson's disease. Mov Disord off J Mov Disord Soc.

[CR11] Raeder V, Boura I, Leta V, Jenner P, Reichmann H, Trenkwalder C, Klingelhoefer L, Chaudhuri KR (2021). Rotigotine transdermal patch for motor and non-motor Parkinson’s disease: a review of 12 years' clinical experience. CNS Drugs.

[CR12] Ray Chaudhuri K, Qamar MA, Rajah T, Loehrer P, Sauerbier A, Odin P, Jenner P (2016). Non-oral dopaminergic therapies for Parkinson's disease: current treatments and the future. NPJ Parkinsons Dis.

[CR13] Rukavina K, Batzu L, Boogers A, Abundes-Corona A, Bruno V, Chaudhuri KR (2021). Non-motor complications in late stage Parkinson's disease: recognition, management and unmet needs. Expert Rev Neurother.

[CR14] Shulman LM, Minagar A, Rabinstein A, Weiner WJ (2000). The use of dopamine agonists in very elderly patients with Parkinson’s disease. Mov Disord.

[CR15] Standaert DG, Aldred J, Anca-Herschkovitsch M, Bourgeois P, Cubo E, Davis TL, Iansek R, Kovács N, Pontieri FE, Siddiqui MS, Simu M, Bergmann L, Kukreja P, Robieson WZ, Chaudhuri KR (2021). DUOGLOBE: one-year outcomes in a real-world study of levodopa carbidopa intestinal gel for Parkinson's disease. Mov Disorde Clin Pract.

[CR16] Timpka J, Henriksen T, Odin P (2016). Non-oral continuous drug delivery techniques in Parkinson's disease: for whom, when, and how?. Mov Clin Pract.

[CR17] Todorova A, Martinez-Martin P, Martin A, Rizos A, Reddy P, Chaudhuri KR (2013). Daytime apomorphine infusion combined with transdermal Rotigotine patch therapy is tolerated at 2 years: A 24-h treatment option in Parkinson’s disease. Basal Ganglia.

[CR18] van Wamelen DJ, Grigoriou S, Chaudhuri KR, Odin P (2018). Continuous drug delivery aiming continuous dopaminergic stimulation in Parkinson's disease. J Parkinsons Dis.

